# Comparative Analysis of *Moringa oleifera* Lam. Leaves Ethanolic Extracts: Effects of Extraction Methods on Phytochemicals, Antioxidant, Antimicrobial, and In Ovo Profile

**DOI:** 10.3390/plants14111653

**Published:** 2025-05-29

**Authors:** Sergio Liga, Ioana Zinuca Magyari-Pavel, Ștefana Avram, Daliana Ionela Minda, Ana-Maria Vlase, Delia Muntean, Laurian Vlase, Elena-Alina Moacă, Corina Danciu

**Affiliations:** 1 Department of Applied Chemistry and Engineering of Organic and Natural Compounds, Faculty of Chemical Engineering, Biotechnologies and Environmental Protection, Politehnica University Timisoara, Vasile Pârvan No. 6, 300223 Timisoara, Romania; sergio.liga96@gmail.com; 2 Department of Pharmacognosy-Phytotherapy, Faculty of Pharmacy, “Victor Babeș” University of Medicine and Pharmacy, Eftimie Murgu Square, No. 2, 300041 Timisoara, Romania; stefana.avram@umft.ro (Ș.A.); corina.danciu@umft.ro (C.D.); 3 Research and Processing Center for Medicinal and Aromatic Plants, “Victor Babeș” University of Medicine and Pharmacy, Eftimie Murgu Square, No. 2, 300041 Timisoara, Romania; 4 Department of Pharmaceutical Botany, Iuliu Hațieganu University of Medicine and Pharmacy, 8 Victor Babeș Street, 400347 Cluj-Napoca, Romania; gheldiu.ana@umfcluj.ro; 5 Multidisciplinary Research Center on Antimicrobial Resistance, Department of Microbiology, Faculty of Medicine, “Victor Babeș” University of Medicine and Pharmacy, Eftimie Murgu Square, No. 2, 300041 Timisoara, Romania; muntean.delia@umft.ro; 6 Department of Pharmaceutical Technology and Biopharmaceutics, University of Medicine and Pharmacy, 8 Victor Babeș Street, 400347 Cluj-Napoca, Romania; laurian.vlase@umfcluj.ro; 7 University Clinic of Toxicology, Drug Industry, Management and Legislation, Faculty of Pharmacy, “Victor Babeș” University of Medicine and Pharmacy, 300041 Timisoara, Romania; alina.moaca@umft.ro; 8 Research Center for Pharmaco-Toxicological Evaluation, “Victor Babeș” University of Medicine and Pharmacy, 300041 Timisoara, Romania

**Keywords:** *Moringa oleifera* Lam., ethanolic extracts, antioxidant, antibacterial, CAM assay

## Abstract

A comparative evaluation of *Moringa oleifera* Lam. ethanolic leaf extracts was performed using different extraction methods (maceration or ultrasound-assisted) and the qualitative and quantitative profile of the bioactive compounds contained were further assessed. The antioxidant potential and antimicrobial activity were evaluated, as well as the antiangiogenic effects through in ovo studies. Six ethanolic extracts were tested in this study. Moringa MAC 70% and Moringa US 70% extracts displayed the highest concentration of phenolic compounds and also showed a significant AOA at the highest tested dose. Regarding the antimicrobial effect, the extracts obtained with 70% ethanol (maceration or ultrasound-assisted) had antimicrobial activity against *S. aureus*, *S. pyogenes* and *E. coli*, followed by *Candida parapsilosis*. On the *Pseudomonas aeruginosa* strain, the extracts showed no effect. The HET-CAM assay showed that the extracts did not cause any irritation compared to the used positive control. Furthermore, the extracts Moringa MAC 70% and Moringa US 70% did not affect the normal process of blood vessel formation. The data obtained highlights that, from the six tested extracts, the ones obtained with 70% ethanol using maceration and ultrasound-assisted methods (Moringa MAC 70% and Moringa US 70%) showed the highest phenolic content and exhibited the strongest antioxidant activity. The same two extracts did not show signs of irritation in the HET-CAM model.

## 1. Introduction

Medical research is being conducted in the development of plant-based products, a significant modern initiative that utilizes the therapeutic properties of bioactive compounds found in plants. The incorporation of plant products into safe drugs has been the subject of many scientific efforts using synthetic or greener strategies [1,2,3].

The demand for herbal alternatives to conventional industrial pharmaceuticals is growing among consumers. This growing interest has resulted in the development of advanced extraction and formulation techniques that enhance the bioavailability of plant extracts [4,5]. Despite this, there are many opposing views on the effectiveness and safety of these plant-based products. The absence of standardized doses and inconsistent quality control are the primary reasons for concern regarding herbal products [6].

*Moringa oleifera* Lam. (abbreviated in this study as MO), commonly known as ‘drumstick tree’ or ‘horseradish tree’, is a plant that belongs to the *Moringaceae* family, *Magnoliopsida* class and subfamily of *Tracheobionta* [7,8,9]. Based on the APG IV criteria, the *Moringaceae* family is now classed as part of the *Brassicales* order [10].

In the scientific literature about 33 species have been reported in the *Moringaceae* family, but only twelve, *M. arborea*, *M. borziana*, *M. concanensis*, *M. drouhardi*, *M. hildebrandtii*, *M. longituba*, *M. oleifera*, *M. ovalifolia*, *M. peregrina*, *M. rivae*, *M. ruspoliana* and *M. stenopetala*, are well-known worldwide [7,8,9,11].

**Figure 1:**
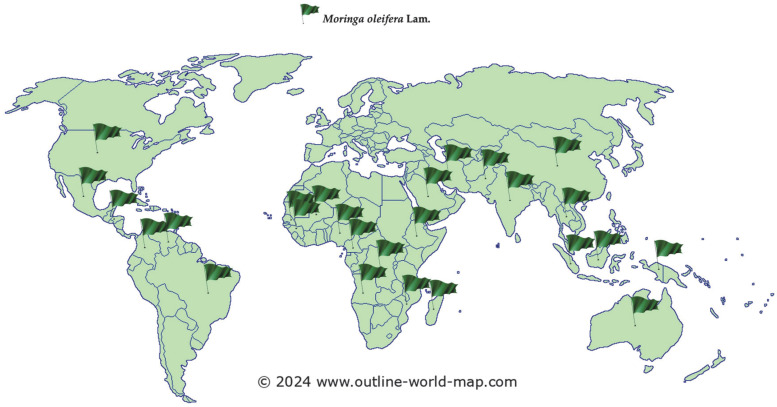
World map distributions of *M. oleifera* Lam. The image was obtained and modified from Outline World Map (free access).

*M. oleifera* Lam. is native to South Asia and is widely distributed in various tropical and arid regions (Figure 1), such as Afghanistan, Pakistan, Senegal, Nigeria, Ethiopia, the Caribbean Islands, Latin America, the Pacific Islands, Australia, Madagascar, and Central America [7,11,12,13,14].

**Figure 2:**
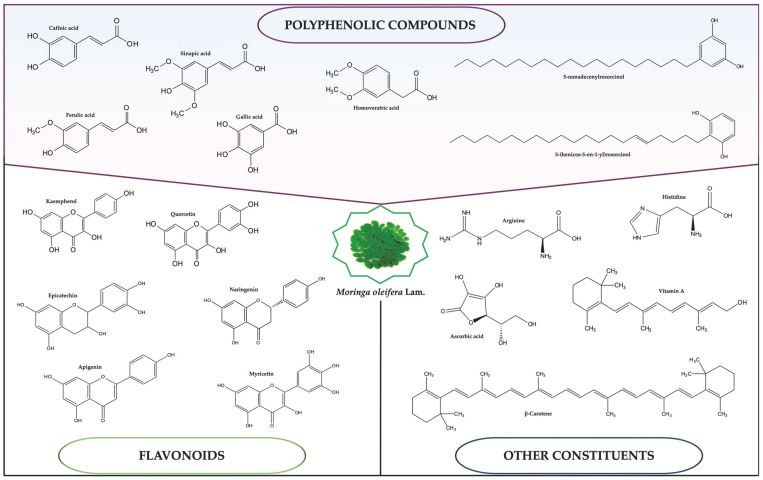
Main bioactive compounds found in *Moringa oleifera* Lam. tree, according to literature.

The use of *M. oleifera* Lam. in traditional folk medicine has resulted in many researchers becoming interested in its therapeutic properties. According to phytochemistry, the Moringa tree’s leaves and seeds contain a significant amount of naturally active compounds that can be beneficial for therapy (Figure 2). These include polyphenolic compounds, flavonoids, vitamins, carotenoids, tannins, saponins, and various metals [15,16,17,18,19,20,21]. These bioactive compounds possess significant therapeutic effects, including antioxidant, antimicrobial, anti-inflammatory, anti-cancer, antiviral, immune boosting, carbohydrate and lipid metabolism regulation, wound repair and healing, intestinal microbiota regulation, protection against aging signs, hypotensive, and other qualities [11,16,21,22,23,24,25,26,27,28,29,30,31,32,33,34,35,36].

In the process of natural product research and drug discovery, extracting bioactive compounds from medicinal plants is a fundamental step. In the past, traditional extraction techniques—maceration, infusion, decoction, percolation—have been widely used due to their simplicity, affordability, and suitability for thermolabile compounds [37,38]. Some of the disadvantages of these traditional methods are their long extraction times, low efficiency, and high solvent consumption. In response, modern extraction techniques (e.g., ultrasound-assisted extraction, microwave-assisted extraction, accelerated solvent extraction) have become increasingly valuable and green bio-refining tools in contemporary phytochemical investigations due to their efficiency, eco-friendliness, and improved yield and resource consumption [5,38,39]. Integrating both methods allows for a robust comparison of extraction performance and compound stability, optimizing the recovery of bioactive metabolites.

Known for its diverse pharmacological properties, MO has been extensively studied for its antioxidant, antimicrobial, anti-inflammatory, and wound-healing activities [9,16,17]. Despite this, there is still a lack of clarity in the literature regarding how different extraction techniques and solvent concentrations influence the phytochemical content and biological activity of its leaf extracts. In particular, the antiangiogenic potential of Moringa remains underexplored, with existing evidence largely limited to in vitro cytotoxicity assays or indirect inflammatory markers [40]. Moreover, comparisons across studies are hindered by the lack of extract standardization and limited integration of biochemical and functional endpoints. Most existing studies either focus on a single method or do not establish direct comparisons between traditional and modern extraction approaches. Moreover, while many studies have demonstrated the biological potential of MO, few have explored the relationship between extraction parameters and specific bioactivities, such as antioxidant, antimicrobial, and antiangiogenic effects. These properties were chosen for the present study due to their relevance in oxidative stress-related diseases, infection management, and tissue regeneration—core aspects of the plant’s traditional use in wound healing and dermatological conditions [16,17]. Additionally, the inclusion of irritancy and angio-inhibitory assessments, particularly using the CAM assay, addresses a critical need for evaluating the safety and functional potential of MO extracts in topical applications and cosmetic development. To address these gaps, a comparative evaluation of MO ethanolic leaves extracts was performed using different extraction methods (traditional extraction technique—maceration, and modern extraction technique—ultrasound-assisted). Different solvents showed different selectivity to the bioactive compounds present in the MO leaves and, by analyzing scientific literature data [41,42,43], choice in this study was primarily focused on a safe, non-toxic, and suitable polar compatibility solvent (e.g., ethanol). By analyzing the phytochemical profiles and assessing key bioactivities, this research aims to provide a deeper understanding of how extraction techniques influence therapeutic efficacy.

The present study aims to investigate the effects of different extraction methods on the chemical composition and biological activities of *Moringa oleifera* leaf extracts—including antioxidant, antimicrobial, irritant, and antiangiogenic effects. This integrated approach provides novel insights into the multi-target potential of Moringa, supporting the development of safe and effective formulations for functional and cosmetic applications.

## 2. Results and Discussion

### 2.1. Identification and Quantification of Phenolic Compounds in Moringa Ethanolic Extracts

Firstly, the total amount of phytocompounds obtained was identified and quantified. As shown in Table 1, besides what was previously known in the scientific literature [44,45,46], the present study has found that all six *Moringa oleifera* Lam. ethanolic extracts contain high concentrations of polyphenolic compounds and flavonoids. The Moringa ethanolic extracts that contain these specific compounds were influenced by the extraction method and parameters, according with previous reports [47,48,49,50,51]. The study also revealed that the concentration of ethanol used plays a significant role in the extraction of polyphenolic compounds.

In this study, the extracts display different amounts of phenolic compounds, with Moringa MAC 70% and Moringa US 70% having a higher content than the other ethanolic extracts. All six extracts contained polyphenols, which were identified as iso-quercitrin, 4-*O*-caffeoylquinic acid and quercetin, with iso-quercitrin being the most abundant among these polyphenols. In some extracts caffeic acid, and p-coumaric acid have been detected in lower concentrations or even trace amounts. In both extracts (Moringa MAC 70% and Moringa US 70%), the concentration of iso-quercitrin was almost equivalent. This finding aligns with previous research; in a study conducted by Wang et al. [52], the bioactive compounds extracted and identified with 70% ethanol were mainly kaempferol and quercetin. Another study, conducted by Erasmo Herman-Lara et al. [53], discovered that the major compounds were two derivatives of quercetin (quercetin-O-hexoside) and neochlorogenic acid, as well as caffeoylquinic acid, and kaempferol derivatives.

The present study evaluated the polyphenolic profile of *Moringa oleifera* Lam. leaf extracts obtained through maceration (MAC) and ultrasound-assisted extraction (US) using ethanol at different concentrations (30%, 50%, and 70%). The results revealed that extraction method and ethanol concentration significantly influenced the recovery of polyphenolic compounds.

US extraction generally led to higher polyphenol yields compared to MAC, particularly for flavonoid glycosides and phenolic acids. The highest concentrations of caffeic acid (0.676 ± 0.067 µg/mL), 4-*O*-caffeoylquinic acid (53.735 ± 5.373 µg/mL), *p*-coumaric acid (1.876 ± 0.056 µg/mL), vitexin (9.504 ± 0.475 µg/mL), rutin (45.631 ± 3.194 µg/mL), and kaempferol (5.813 ± 0.523 µg/mL) were observed in US extracts. These results suggest that ultrasound waves propagate through the extraction medium, generating cavitation effects that help disrupt the leaf cell wall, thereby enhancing the release of phytochemicals from the plant matrix during the extraction process [54]. Similar trends were observed in previous studies, where ultrasound-assisted extraction enhanced flavonoid recovery from *Moringa oleifera* Lam. leaves compared to conventional methods [55,56,57].

The maceration technique relies on diffusion and osmosis to facilitate the release of phytochemicals from the softened plant cell walls. This method typically requires longer extraction times to achieve a high yield of total phenolic content [58]. In this study, MAC and US extraction techniques yielded comparable overall polyphenol recovery from *Moringa oleifera* Lam. leaves. The highest concentration of chlorogenic acid (2.994 ± 0.179 µg/mL) and quercitrin (68.703 ± 8.931 µg/mL) were obtained with MAC EtOH70%, whereas the highest amount of quercetin (7.299 ± 1.021 µg/mL) was obtained with MAC EtOH30%. However, for iso-quercitrin, the concentrations were almost equivalent between MAC EtOH70% and US EtOH70% (223.323 ± 11.166 µg/mL versus 223.015 ± 33.452 µg/mL).

Solvent concentration played a crucial role in extracting specific polyphenols. Higher ethanol concentrations (50% and 70%) significantly increased the extraction of quercetin glycosides, rutin, and caffeoylquinic acid, compounds known for their antioxidant and anti-inflammatory properties. The highest amounts of 4-O-caffeoylquinic acid (53.735 ± 5.373 µg/mL) were detected in US EtOH 70%, while *p*-coumaric acid was found in small amounts in MAC EtOH 30%, US EtOH 30% and 70%, respectively.

### 2.2. Qualitative Characterization Technique (ATR FT–IR) for MO Ethanolic Extracts

The qualitative characterization of the six ethanolic extracts of MO was established using the ATR FTI–R technique, following the identification and quantification of polyphenolic compounds.

**Figure 3:**
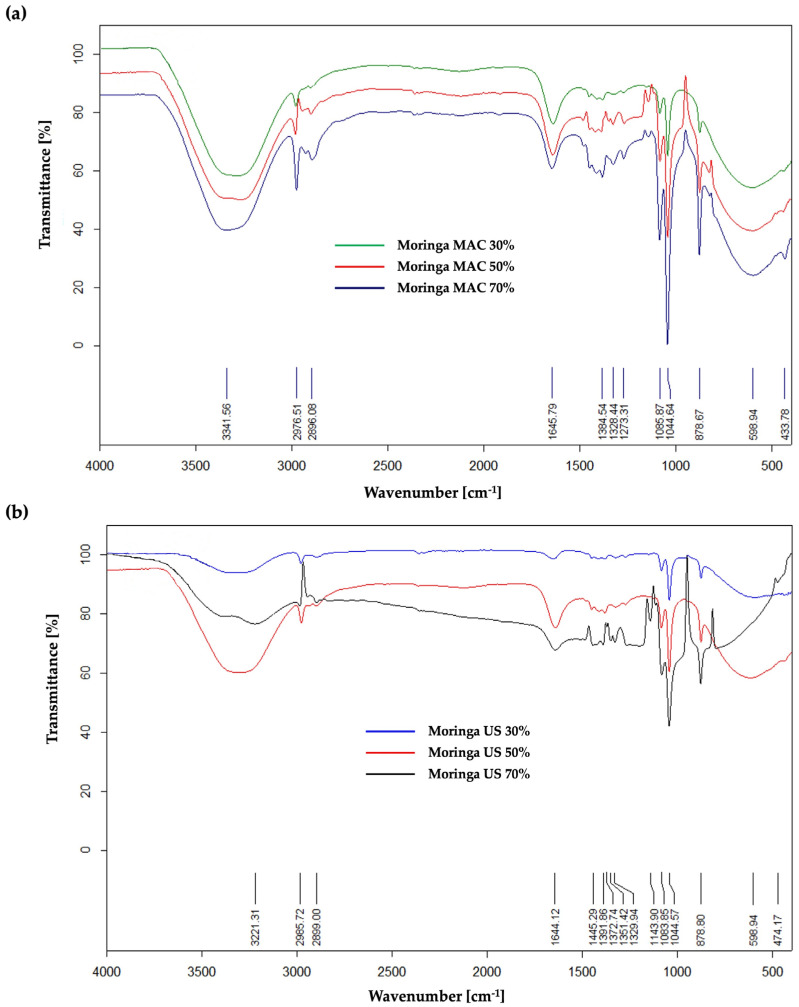
ATR FT–IR spectrum of Moringa MAC (**a**) and Moringa US (**b**).

In the case of the extracts obtained by maceration process (Figure 3a), the ATR FT–IR spectrum revealed significant absorption peaks, which will be further described in detail. Functional groups of O-H stretch vibrations from polyphenolic compounds are represented by the strong broadband at wavelength 3341.56 cm^−1^, while those from carboxylic acids or alcohols contained in the extracts have absorption peaks around 2976.51 cm^−1^ and 2896.08 cm^−1^. The peak recorded at 1645.79 cm^−1^ could be attributed to C=O functional groups found in aldehydes, esters, aliphatic ketones or carboxylic acids. Additionally, at the wavelength 1384.54 cm^−1^, both absorption peaks of functional C-H groups of alkanes or aldehydes appeared, as well as absorption peaks of functional O-H groups from alcohols or phenols. Absorption peaks recorded at 1044.64 cm^−1^ and 1085.87 cm^−1^ could be attributed to functional C-N groups from amines or functional CO-O-CO vibration groups from anhydrides. The remaining recorded absorption peaks under 1000 cm^−1^ (878.67 cm^−1^, 598.94 cm^−1^, and 433.78 cm^−1^) are attributed to the spectral prints (bending vibration from aromatics alkenes =C-H). Based on the obtained spectra, it can be determined that the absorption bands are more prominent in the case of Moringa MAC 70%, which means that the extraction yield is higher.

In the case of MO ethanolic extracts obtained by ultrasound-assisted extraction, the spectrum (Figure 3b) revealed important absorption peaks at 3221.31 cm^−1^, 2985.72 cm^−1^, 2899.0 cm^−1^, 1644.12 cm^−1^, 1445.29 cm^−1^, 1391.86 cm^−1^, 1083.85 cm^−1^, and 1044.57 cm^−1^. It has been confirmed once more that the extraction of bioactive compounds from MO is influenced by the extraction method and parameters.

### 2.3. Antioxidant Activity of MO Ethanolic Extracts Using DPPH Radical Scavenging Assay

**Figure 4:**
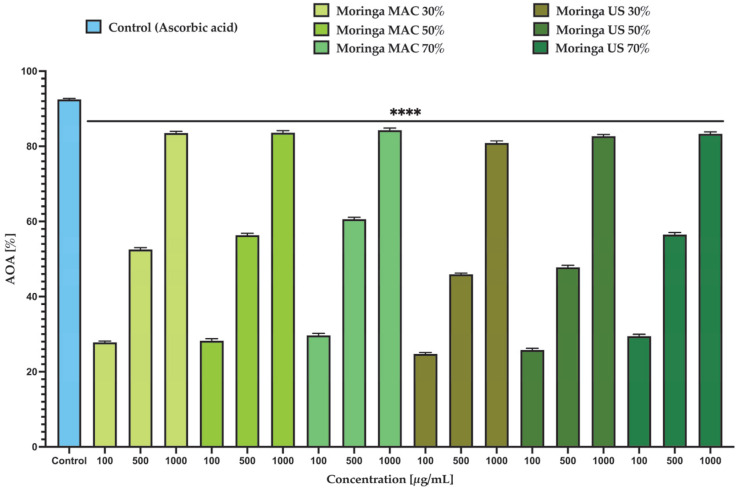
Antioxidant activity of MO ethanolic extracts. The data are expressed as mean ± SD. One-way ANOVA test followed by a Dunnett’s multiple comparison test was used to compare groups (**** *p* < 0.0001).

Figure 4 represents the MO ethanolic extracts’ AOA [%] at 100 and 1000 µg/mL using the DPPH assay, compared to the standard ascorbic acid (100 µg/mL). The results revealed that the highest AOA was displayed by the Moringa MAC 70%, the value being slightly higher than Moringa US 70% (at 1000 µg/mL; the AOA for Moringa MAC 70% was 84.23%, and for Moringa US 70% was 83.15%), with both antioxidant activities being close to that of ascorbic acid. A one-way ANOVA test was used for the evaluation of the data from Figure 4, and the statistical significance (*p* < 0.0001) presents the differences between the two extracts in terms of their antioxidant activity.

Consistent with our investigation, numerous studies have demonstrated that ethanolic extracts of MO leaves have a greater antioxidant activity, as assessed by the DPPH assay. For example, Oluewu et al. [48] conducted an evaluation of methanolic and ethanolic extracts from five countries and concluded that ethanolic extracts of Moringa leaves have greater DPPH radical scavenging activity than methanolic extracts (e.g., Nigeria: 104.10 vs. 84.94 µmol TE g^−1^; Ghana: 109.35 vs. 86.69 µmol TE g^−1^; India: 112.24 vs. 89.65 µmol TE g^−1^; Haiti: 117.52 vs. 94.83 µmol TE g^−1^; USA: 123.48 vs. 90.71 µmol TE g^−1^). According to another article by Erasmo et al., the total phenolic content is linked to the antioxidant activity, and the 70% ethanolic extracts of Moringa had high antioxidant values (ABTS 0.52 mmol TEAC/g and DPPH 0.46 mmol TEAC/g) [53].

### 2.4. Antimicrobial Activity of Moringa MAC and Moringa US Extracts

The antimicrobial potential of MO ethanolic extracts was evaluated by determining the MIC, MBC and MFC, using the broth dilution method on four different bacterial strains: (i) two Gram-negative bacteria (*Escherichia coli*, *Pseudomonas aeruginosa*); (ii) two Gram-positive bacteria (*Staphylococcus aureus*, *Streptococcus pyogenes*); and (iii) one yeast strain (*Candida parapsilosis*).

According to the data obtained (Table 2), all six extracts exhibited antibacterial activity. The ethanolic extracts obtained with 70% ethanol exhibited antimicrobial activity against *S. aureus*, *S. pyogenes* and *E. coli* (MIC = 12.5 mg/mL), followed by yeast *Candida parapsilosis* (MIC = 25 mg/mL). The extracts that were obtained using ultrasonication also had antibacterial activity against *S. pyogenes* (MIC = 12.5 mg/mL). The MIC and MBC values could not be determined for the *Pseudomonas aeruginosa* strain.

Consistent with our antimicrobial results, Jahan et al. [59] studied the antimicrobial activity of Moringa leaves ethanolic extract on foodborne pathogens. The extract proved to be highly effective in treating *Staphylococcus aureus* and *Escherichia coli* (e.g., MIC = 400 μg/mL, respectively, 500 μg/mL). In a separate study, Enerijiofi et al. [60] investigated the impact of aqueous and ethanolic Moringa leaf extracts on selected clinical bacterial isolates. The results showed that the ethanolic extract had the highest zone of inhibition at 200 mg/mL of 23  ±  0.02 mm for *Staphylococcus aureus*, 25  ±  0.51 for *Pseudomonas aeruginosa*, and 22  ±  0.48 mm for *Escherichia coli*. These studies emphasize the importance of identifying phenolic compounds from *Moringa oleifera* Lam. extracts when assessing their antimicrobial potential against different bacterial strains.

### 2.5. In Ovo Safety Profile of MO Ethanolic Extracts

**Figure 5:**
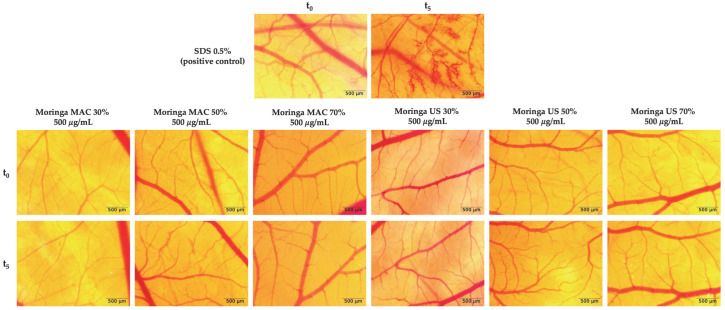
Stereomicroscopic images showing the effects of MO ethanolic extracts on the HET-CAM assay. The images were captured at the initial moment (t_0_) and 5 min (t_5_) post-application of the positive control (SDS 0.5%) or of the extracts (500 µg/mL); scale bars represent 500 µm.

The in ovo evaluation of the potential irritative effect was performed using the HET–CAM method, by calculating the irritation score (IS) for the control group and extracts. According to Figure 5 and Table 3, all five extracts were found to be non-irritants, compared to the positive control (SDS 0.5%), which exhibited a significant irritating effect (IS = 17.29 ± 0.16).

**Figure 6:**
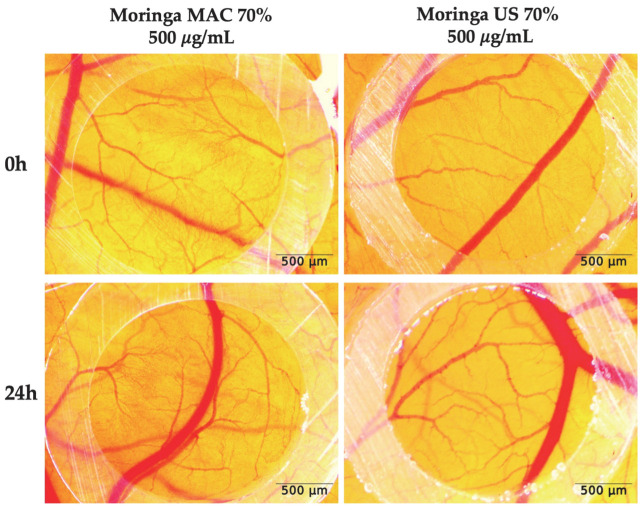
Stereomicroscopic images of Moringa MAC 70% and Moringa US 70% effects on CAM assay. Images were taken initially at 0, and 24 h post-treatment; scale bars represent 500 µm.

Based on the preliminary findings from our study, it can be concluded that the extracts with the highest concentration of extracted bioactive compounds should be further tested, specifically Moringa MAC 70% and Moringa US 70%, for the experimental part of the modulating angiogenesis. The selected extracts were applied to evaluate their potential effects on the active angiogenic process occurring in the developing chorioallantoic membrane from day 7 of incubation, a stage characterized by pronounced vascular development. The extracts were tested at a concentration of 500 µg/mL, which had previously been shown to be well-tolerated without causing any irritant effects on the CAM tissues. The normal process of blood vessel formation during this stage of development was not altered by either of the two extracts (Moringa MAC 70%, Moringa US 70%), supporting normal angiogenic progression, as illustrated in Figure 6.

Angiogenesis is the process of new blood vessel formation, essential for tissue repair, growth, regeneration or, conversely, tumor progression, as it supports cancer cell survival and metastasis [61]. The impact of MO leaf extracts on angiogenesis has been explored in several studies, with contrasting outcomes.

A research paper published by Batmomolin et al. in a rat model of preeclampsia. showed that the administration of MO leaf 905 ethanolic extract led to a trend of decreased serum levels of soluble vascular endothelial growth factor receptor 1 (sFlt-1), a marker associated with impaired angiogenesis [62]. As reported by Al-Ghanayem et al., MO methanolic leaf extract also increased vascular endothelial growth factor (VEGF) expression in HaCaT cells, indicating enhanced angiogenesis that might have contributed to its wound-healing action [63]. Other studies investigated the anti-angiogenic activity of MO. An ethanolic leaf extract was evaluated using the chorioallantoic membrane (CAM) assay, indicating no new blood vessel formation in the treated groups, suggesting potential anti-angiogenic properties of the extract [64]. Another CAM assay study found that methanolic and aqueous MO leaf extracts reduced blood vessel formation in a dose-dependent manner, with the 100% aqueous extract showing the strongest anti-angiogenic activity, surpassing sunitinib [40].

The effects of MO leaf extracts on angiogenesis appear to be context-dependent, exhibiting both inhibitory and stimulatory properties based on concentration, extract preparation, and biological model used. The observed dual effects emphasize the necessity for further research to clarify the mechanisms by which MO influences angiogenesis, considering factors such as extract type concentration, plant part used, and the specific biological model or pathological context.

## 3. Materials and Methods

### 3.1. Plant Materials and Extraction Protocol

The dry plant material used in this study was purchased from a store located in Oradea, Romania (country of origin: India). MO leaves were assigned the voucher number MO1/2024. Two extraction methods were employed to extract the bioactive natural compounds from the leaves of the *M. oleifera* Lam. tree: maceration and ultrasound-assisted extraction. The plant material was dried in a hot air oven at 60 °C for 3 h, followed by recording the final weight (moisture content = 0.082%), and then ground into powder using an electric mill before the extraction process. For the maceration extraction technique, different concentrations of ethanol (30%, 50%, and 70% (*v*/*v*)) were poured over the ground plant material (10 g dry weight/100 mL) and then left in contact for 10 days at room temperature (25 °C). To ensure full extraction, the maceration samples were stirred and shaken regularly. For the ultrasound-assisted extraction, the same amount of ground plant product (10 g dry weight/100 mL) and, respectively, the same concentrations of ethanol (30%, 50%, 70% (*v*/*v*)), were subjected to the sonication process (80% amplitude) for 15 min, using an Ultrasonic Homogenizer UP100H (Teltow, Germany) at room temperature (25 °C).

After that, all ethanolic extracts were filtered using a Whatman filter No. 1 and concentrated to dryness in a rotary vacuum evaporator (Heidolph Laborota 4000, Schwalbach, Germany) at 50 °C. The dried extracts were kept at a temperature below −20 °C before being used for further experiments.

### 3.2. Chemicals and Reagents

The chemicals and reagents used for obtaining the extracts ethanol 96% (*v*/*v*), 2,2-diphenyl-1-picrylhydrazyl (DPPH), and all standards used for LC–MS analysis were purchased from Sigma Aldricht (Schnelldorf, Germany).

### 3.3. Phytochemical Profile of Moringa oleifera Lam. Ethanolic Extracts

#### 3.3.1. HPLC/LC–MS Identification and Quantification of Polyphenolic Compounds

The phytochemical composition of the vegetal extracts was analyzed using LC–MS/MS, applying two previously validated methods. The analyses were conducted on an Agilent 1100 HPLC Series system (Agilent, Santa Clara, CA, USA) coupled with an Agilent Ion Trap 1100 SL mass spectrometer (LC/MSD Ion Trap VL) [45,46,47].

The first LC–MS method was developed for the identification and quantification of polyphenolic compounds, using a Zorbax SB-C18 column (100 mm × 3.0 mm, 3.5 μm) with a methanol/0.1% acetic acid mobile phase in a binary gradient. This method included 28 analytical standards, but only several polyphenols were identified and quantified in the tested extracts, as reported in the Results section. The column was maintained at 48 °C, with a flow rate of 1 mL/min and an injection volume of 5 μL. UV detection was set at 330 nm for polyphenolic acids and 370 nm for flavonoids, while MS detection operated in negative ESI mode [65,66,67]. A second LC–MS method was developed to identify eight additional polyphenols: epicatechin, catechin, syringic acid, gallic acid, vanillic acid, protocatechuic acid, epigallocatechin, and epigallocatechin gallate. The analysis employed the same chromatographic column and instrumentation, with a modified gradient elution. Bioactive compound detection was performed in MS mode under the same ESI conditions [65,66,67].

Identification was achieved by comparing MS spectra and chromatographic traces with library standards, while quantification relied on UV detection and calibration curves from analytical standards. Chromatographic data were processed using DataAnalysis (v5.3) and ChemStation (vB01.03) software (Agilent, Santa Clara, CA, USA). Results were expressed as micrograms of bioactive compound per milliliter of extract.

#### 3.3.2. Fourier Transform–Infrared Spectroscopy (FT–IR) Characterization Technique

The functional groups of MO ethanolic extracts were characterized by FT–IR spectroscopy using a Bruker Vertex 70 spectrophotometer (Bruker Daltonik GmbH, Bremen, Bremen, Germany) equipped with an ATR Platinum module, Bruker Diamond Type A225/Q.I. In the range of 4000 ÷ 400 cm^−1^, approximately 128 scans were co-added for every sample analyzed.

### 3.4. Antioxidant Activity—2,2-Diphenyl-1-Picrylhydrazyl (DPPH) Assay

The DPPH method was performed as previously described [68], with slight modifications to determine the MO extracts’ antioxidant activity (AOA).

Briefly, 0.2 mL of the MO ethanolic extracts (100–1000 µg/mL) was mixed with 1.8 mL of 0.1 mM DPPH prepared in ethanol and incubated for 30 min at room temperature in the dark. Ascorbic acid was used as control. The absorbance was measured at 517 nm employing a UV–VIS spectrophotometer (PG Instruments Ltd., Lutterworth, UK). The AOA (%) was calculated by the following formula:

(1) $$AOA\;\left(\%\right)=\left[\frac{A_{0\;(control)}-A_{sample}}{A_{0\;(control)}}\right]\times 100$$

### 3.5. Antimicrobial Potential

The MO ethanolic extracts were evaluated for their antimicrobial activity against five selected pathogenic microbial strains (Thermo Scientific, Waltham, MA, USA), used for testing: *Staphylococcus aureus* ATCC 25923, *Streptococcus pyogenes* ATCC 19615, *Escherichia coli* ATCC 25922, *Pseudomonas aeruginosa* ATCC 27853, and *Candida parapsilosis* ATCC 22019.

The antimicrobial activity was evaluated in accordance with the recommendations set forth by the European Committee on Antimicrobial Susceptibility Testing (EUCAST) [69] and the Clinical Laboratory Standard Institute (CLSI) [70], also used in our previous studies [71,72]. All pathogenic microbial strains were isolated on Columbia agar with 5% sheep blood, while Sabouraud agar with chloramphenicol was utilized for yeast *Candida parapsilosis*. The microbial suspensions with 0.85% NaCl were used to achieve a concentration of 0.5 McFarland. The minimum inhibitory (MIC), bactericidal (MBC), or fungicidal (MFC) concentrations were determined by broth dilution assay using microbial suspensions cultured on Mueller–Hinton agar/Mueller–Hinton fastidious agar.

### 3.6. In Ovo CAM Assay

The ethanolic extracts of MO were subjected to in ovo evaluation to determine their irritant potential and to explore their possible modulatory effects on angiogenesis. The in ovo chorioallantoic membrane (CAM) assay was conducted as previously described [73,74,75]. The standard procedure involves incubating fertilized chicken eggs (*Gallus gallus domesticus*) at 37 °C in a humidified environment. On day 3, a defined volume of albumen is removed, and the upper eggshell is carefully opened and resealed. Incubation continues until day 7, when the experimental procedures are performed.

The potential irritant effect of MO ethanolic extract administration upon the CAM was assessed using the HET-CAM protocol [76], with minor modifications adapted to our laboratory conditions [74]. The irritant potential of the extracts was evaluated using the chorioallantoic membrane (CAM) assay on fertilized chicken eggs. Following sample application, vascular responses—hemorrhage (H), coagulation (C), and lysis (L)—were observed immediately under a stereomicroscope. Under these conditions, seven samples were tested: (1) positive control, represented by sodium dodecyl sulfate (SDS) 0.5%; (2) Moringa maceration ethanolic extract 30% (Moringa MAC 30%); (3) Moringa maceration ethanolic extract 50% (Moringa MAC 50%); (4) Moringa maceration ethanolic extract 70% (Moringa MAC 70%); (5) Moringa ultrasound-assisted ethanolic extract 30% (Moringa US 30%); (6) Moringa ultrasound-assisted ethanolic extract 50% (Moringa US 50%); and (7) Moringa ultrasound-assisted ethanolic extract 70% (Moringa US 70%). The tested extracts applied on the membranes were evaluated stereomicroscopic over a period of 300 s. The irritation score (IS) was calculated using the following equation (Equation (2)) and interpreted according to the Luepke scale [77]:

(2) $$IS=5\times \frac{301-{Sec}_{H}}{300}+7\times \frac{301-{Sec}_{L}}{300}+9\times \frac{301-{Sec}_{C}}{300}$$

To investigate the effect of MO ethanolic extracts on angiogenesis, samples (500 μg/mL in 0.5% DMSO) were applied in 5 μL volumes onto the CAM surface on day 7 of incubation, using plastic rings (3 mm in diameter) previously positioned on the membrane.

All tested extracts were monitored daily using a stereomicroscope (ZEISS SteREO Discovery. V8, Göttingen, Germany). Stereomicroscopic images were captured and processed with the Axiocam 105 color camera and AxioVision SE64 Rel. 4.9.1 software (ZEISS, Göttingen, Germany), with further image analysis conducted using ImageJ software (version 1.53k; https://imagej.nih.gov/ij/index.html, accessed on 9 July 2024). The experiment was performed in triplicate.

### 3.7. Statistical Analysis

The data obtained in the present study are expressed as mean ± standard deviation (SD). A one-way ANOVA test followed by a Dunnett’s multiple comparison test was used to compare groups. Difference showing a *p* level of 0.05 or lower was considered to have statistical significance. GraphPad Prism 10.4.1 (GraphPad Software, San Diego, CA, USA) was used for the statistical analysis.

## 4. Conclusions

The findings of the present study provide a broader evaluation of *Moringa oleifera* leaf ethanolic extracts, demonstrating that both the extraction method and solvent concentration significantly influence the phytochemical composition and potential biological activities of the samples. The Moringa MAC 70% and Moringa US 70% extracts contained the highest levels of phenolic compounds, particularly iso-quercitrin, 4-O-caffeoylquinic acid, and quercetin. These extracts also exhibited the most pronounced antioxidant activity. The extracts obtained with 70% ethanol, regardless of the extraction method used, showed antimicrobial activity against *S. aureus*, *S. pyogenes* and *E. coli* followed by the yeast *Candida parapsilosis*. On the HET-CAM assay, the extracts did not cause any irritation compared to the positive control used. Furthermore, the extracts Moringa MAC 70% and Moringa US 70% did not affect the normal process of blood vessel formation. This study supports the practical application of *Moringa oleifera* leaf extracts as natural antioxidants and antimicrobials in health-related products. The high phenolic content and significant bioactivity suggest potential uses in functional foods, cosmetics, and topical treatments. Their safety profile and effectiveness, combined with eco-friendly extraction methods, further highlight their value for sustainable pharmaceutical and nutraceutical development.

## Figures and Tables

**Table 1:** Identification and Quantification of Polyphenolic Compounds by HPLC/LC–MS.

No.	Compounds	Moringa MAC EtOH30%	Moringa MAC EtOH50%	Moringa MAC EtOH70%	Moringa US EtOH30%	Moringa US EtOH50%	Moringa US EtOH70%
		Results (μg/mL)					
1	Gentisic acid	<LOQ	<LOQ	<LOQ	<LOQ	<LOQ	ND
2	Caffeic acid	<LOQ	<LOQ	<LOQ	<LOQ	<LOQ	0.676 ± 0.067
3	Chlorogenic acid	<LOQ	2.768 ± 0.083	2.994 ± 0.179	<LOQ	1.786 ± 0.053	1.484 ± 0.074
4	4-*O*-caffeoylquinic acid	38.582 ± 5.787	35.865 ± 2.511	48.148 ± 4.814	31.043 ± 4.656	38.849 ± 3.107	53.735 ± 5.373
5	*p*-coumaric acid	0.612 ± 0.048	<LOQ	<LOQ	1.876 ± 0.056	<LOQ	0.521 ± 0.067
6	Ferulic acid	<LOQ	ND	ND	<LOQ	ND	ND
7	Vitexin	8.418 ± 0.336	6.449 ± 0.709	8.961 ± 0.806	6.992 ± 0.699	7.739 ± 1.006	9.504 ± 0.475
8	Iso-quercitrin	186.491 ± 7.459	161.603 ± 16.161	223.323 ± 11.166	126.235 ± 13.885	151.4312 ± 9.085	223.015 ± 33.452
9	Rutin	31.454 ± 1.258	28.634 ± 2.004	40.287 ± 4.028	28.337 ± 2.833	32.791 ± 1.967	45.631 ± 3.194
10	Quercitrin	62.159 ± 7.459	50.568 ± 2.528	68.703 ± 8.931	41.594 ± 3.327	42.716 ± 6.407	66.085 ± 0.661
11	Quercetin	7.299 ± 1.021	1.904 ± 0.266	2.482 ± 0.248	17.787 ± 2.312	1.738 ± 0.173	2.206 ± 0.242
12	Luteolin	ND	ND	ND	ND	ND	<LOQ
13	Kaempferol	3.691 ± 0.147	1.037 ± 0.021	1.037 ± 0.134	5.813 ± 0.523	0.672 ± 0.006	<LOQ
14	Apigenin	<LOQ	ND	ND	<LOQ	ND	ND
15	Epicatechin	0.156 ± 0.012	0.101 ± 0.014	0.098 ± 0.002	0.149 ± 0.007	0.152 ± 0.016	0.184 ± 0.005
16	Catechin	ND	0.007 ± 0.001	0.021 ± 0.001	ND	0.012 ± 0.001	ND
17	Gallic acid	0.298 ± 0.041	0.223 ± 0.008	0.348 ± 0.031	0.296 ± 0.005	0.293 ± 0.002	0.497 ± 0.069
18	Protocatechuic acid	0.828 ± 0.091	0.5 ± 0.075	0.628 ± 0.069	0.694 ± 0.062	0.532 ± 0.005	0.386 ± 0.007
19	Epigallocatechin	0.41 ± 0.024	0.199 ± 0.013	0.151 ± 0.015	0.236 ± 0.007	0.145 ± 0.021	0.147 ± 0.011

tOH—ethanol; <LOQ (below limit of quantification); ND—not detected; Concentrations are expressed as mean ± SD (*n* = 3).

**Table 2:** The Minimal Inhibitory Concentration (MIC), Minimal Bactericidal Concentration (MBC), and Minimal Fungal Concentration (MFC) of MO ethanolic extracts for the tested strains.

Bacterial and Yeast Strains	Ethanolic Extracts	MIC Value (mg/mL)	MBC/MFC (mg/mL)
Staphylococcus aureus ATCC 25923	Moringa MAC 30%	25	25
	Moringa MAC 50%	25	25
	Moringa MAC 70%	12.5	12.5
	Moringa US 30%	25	25
	Moringa US 50%	25	25
	Moringa US 70%	12.5	12.5
Streptococcus pyogenes ATCC 19615	Moringa MAC 30%	25	25
	Moringa MAC 50%	25	25
	Moringa MAC 70%	12.5	12.5
	Moringa US 30%	12.5	12.5
	Moringa US 50%	12.5	12.5
	Moringa US 70%	12.5	12.5
Escherichia coli ATCC 25922	Moringa MAC 30%	25	25
	Moringa MAC 50%	25	25
	Moringa MAC 70%	12.5	12.5
	Moringa US 30%	25	25
	Moringa US 50%	25	25
	Moringa US 70%	12.5	12.5
Pseudomonas aeruginosa ATCC 27853	Moringa MAC 30%	* NA	* NA
	Moringa MAC 50%		
	Moringa MAC 70%		
	Moringa US 30%	* NA	* NA
	Moringa US 50%		
	Moringa US 70%		
Candida parapsilosis ATCC 22019	Moringa MAC 30%	50	50
	Moringa MAC 50%	50	50
	Moringa MAC 70%	25	25
	Moringa US 30%	25	25
	Moringa US 50%	25	25
	Moringa US 70%	25	25

* NA—no activity.

**Table 3:** Irritability evaluation using the HET-CAM assay for MO ethanolic extracts (in concentration of 500 µg/mL) and positive control sample (SDS 0.5%).

Test Compound and Controls	Irritation Score (IS)	* Irritation Category
**Positive control: SDS 0.5%**	17.29 ± 0.16	Strong irritant
**Moringa MAC 30%, 500 μg/mL**	0 ± 0.0	Non-irritant
**Moringa MAC 50%, 500 μg/mL**	0 ± 0.0	Non-irritant
**Moringa MAC 70%, 500 μg/mL**	0 ± 0.0	Non-irritant
**Moringa US 30%, 500 μg/mL**	0 ± 0.0	Non-irritant
**Moringa US 50%, 500 μg/mL**	0 ± 0.0	Non-irritant
**Moringa US 70%, 500 μg/mL**	0 ± 0.0	Non-irritant

* Luepke scale: non-irritant (0 ÷ 0.9), weak irritant (1 ÷ 4.9), moderate irritant (5 ÷ 8.9), strong irritant (8.9 ÷ 21).

## Data Availability

Data are contained within the article.
